# Gender Matters: The Relationship between Social Anxiety and Alcohol-Related Consequences

**DOI:** 10.1371/journal.pone.0115361

**Published:** 2014-12-26

**Authors:** Amie R. Schry, Melissa M. Norberg, Brenna B. Maddox, Susan W. White

**Affiliations:** 1 Department of Psychology, Virginia Tech, Blacksburg, Virginia, United States of America; 2 Department of Psychology, Macquarie University, Sydney, New South Wales, Australia; University of Ariel, Israel

## Abstract

**Background and Objectives:**

Identification of risk factors for alcohol-related consequences is an important public health concern. Both gender and social anxiety have been associated with alcohol-related consequences broadly, but it is unknown whether these variables are differentially related to specific types of alcohol-related consequences for American college students.

**Methods:**

In the present study, 573 undergraduate students (*M*_age_ = 19.86 years, *SD* = 1.40; range 18 to 25; 68.9% female) completed an on-line assessment of social anxiety, alcohol use, and four types of alcohol-related consequences (personal, social, physical, and role). Poisson regressions were run to examine social anxiety, gender, and the interaction between social anxiety and gender as predictors of each type of alcohol-related consequences.

**Results:**

After controlling for alcohol use, social anxiety was positively associated with all four types of consequences, and females endorsed higher rates of physical, personal, and role consequences. The interaction between social anxiety and gender was statistically significant only for physical consequences, with social anxiety having a stronger effect for males.

**Discussion and Conclusions:**

These findings, which diverge somewhat from those of a prior study with Australian college students, are discussed in the context of a biopsychosocial model of social anxiety and substance use problems.

**Scientific Significance:**

This study highlights the importance of further investigating cultural differences in the relationships among social anxiety, gender, and alcohol-related consequences.

## Introduction

Alcohol use can lead to a wide range of consequences, including engagement in risky behavior, missing work or school, and experiencing hangovers, memory loss, injuries, and legal problems [1], [2]. Although alcohol consumption is a prerequisite for aversive alcohol-related consequences (ARCs), it does not fully explain the extent of ARCs experienced by university students [3]. Hence, researchers have been concerned with identifying other factors that place university students at risk for ARCs.

Social anxiety is one factor that may contribute to students' experience of ARCs. Although social anxiety is negatively associated with quantity as well as frequency of alcohol use [4], both subclinical social anxiety and social anxiety disorder (SAD) have been shown to almost double university students' risk for an alcohol use disorder [5]. According to the biopsychosocial model of social anxiety and substance use, individuals with social anxiety are prone to experience ARCs, despite drinking less, due to the very nature of social anxiety [6]. Socially anxious individuals are thought to use alcohol to decrease negative affect, increase positive affect, and avoid social scrutiny rather than use more adaptive coping skills in social situations [6]. In support of this assumption, one study found that drinking to cope with social situations (and avoiding these situations when alcohol is unavailable) mediated the relationship between social anxiety and ARCs [7].

Gender (or sex) is another factor that may contribute to students' experience of ARCs. Although sex connotes a biological distinction and gender is socially constructed, the terms have largely been used interchangeably in the extant literature. Therefore, studies examining both gender and sex differences were reviewed. To date, research suggests that men and women may experience certain types of ARCs at different rates. Multiple studies have shown that male college students more frequently miss class, fight, steal, get into trouble with police, and require medical attention due to excessive alcohol consumption than female college students [3], [8], [9]. In contrast, while some studies have shown that college women are more likely to report problems at home, work, or school due to their alcohol use than are men [10], other research has shown no gender difference in rates of these ARCs [11]. Additionally, a recent Australian study suggests that undergraduate women may be more likely to experience physical ARCs, such as hangovers and blackouts than undergraduate men [8], but American research has not supported this finding [11]. As a whole, these findings suggest that undergraduate men may be more likely than undergraduate women to experience acute ARCs related to deviant behavior, public risk-taking, and personal injury, but that other types of ARCs may be experienced equally by both American male and female college students [11].

To date, only one study has assessed if social anxiety and gender differentially affect the type of ARCs that are experienced among university students. Using the modified Timeline Followback (mTLFB) [1] and a sample of university students in Australia, Norberg and colleagues [12] found that socially anxious female students reported more ARCs related to role functioning (e.g., school and work impairments, neglecting responsibilities) than men high or low in social anxiety, and more personal consequences (e.g., putting self in risky situations, feeling bad about drinking) than female students low in social anxiety, when controlling for alcohol consumption. Women, irrespective of social anxiety, reported more physical consequences (e.g., passing out, hangovers) than men. Men with low to moderate social anxiety reported more social consequences (e.g., having a verbal argument, having a physical conflict) than women with low to moderate social anxiety or women and men high in social anxiety.

We are uncertain if the original findings of Norberg and colleagues [12] apply to college students in the United States. It is possible that underage drinking influences findings from research using college student samples cross-nationally, given that the legal drinking age varies across countries (e.g., 18 years in Australia, 21 years in the United States [13]). Only 1.7% of the participants in Norberg and colleagues' study [12] were under the legal drinking age, whereas many college students in the United States are below the legal drinking age [14]. Underage drinkers may experience different consequences relative to peers who are of legal age, given the former typically drink in places that do not require proof of age, such as in homes, at parks, and on beaches [15]. Additionally, raising the legal drinking age in the United States resulted in a 29% reduction in alcohol-related traffic fatalities between 1982 and 2010, which suggests that legality of alcohol use may be associated with driving under the influence and possibly other ARCs amongst young people [16]. Although rates of alcohol dependence among community members do not differ between these countries [17], it remains unknown whether differences in acute ARCs exist amongst university students.

The purpose of this study was to replicate Norberg and colleagues' study [12] with a sample of university students in the United States. We hypothesized that females high in social anxiety would report more personal and role ARCs than females with low to moderate social anxiety. We also expected that males would report more social ARCs (e.g., arguments, physical fights, and damaging property) than females. No a priori hypotheses about physical ARCs were proposed due to contradictory findings in previous studies [8], [11], [12].

## Materials and Methods

### Participants

The sample (*n* = 853) was recruited at a large public university in the Southeastern United States. We excluded 183 (21.5%) participants who reported not consuming alcohol in the 30 days prior to completing the survey. Another 97 participants were excluded for invalid data based on answering at least one reading validity item incorrectly (e.g., when presented with the item, “For this item, please choose 3,” they chose any response option other than 3). Therefore, the final sample on which analyses were run included 573 undergraduate students (*M*_age_ = 19.86 years, *SD* = 1.40; range 18 to 25). This sample was also used in a study examining the factor structure of the mTLFB [18].

The majority of the sample was female (68.9%). More than two-thirds (68.2%) of participants were younger than the legal drinking age in the United States (i.e., younger than 21 years old). Most participants (86.6%) were white, with the next most frequently endorsed race/ethnicity (participants could select multiple races/ethnicities) being Asian/Asian-American (7.0%), which is consistent with university demographics [19]. Participants were asked to report the college at the university in which their major(s) were housed, but they were not asked to report their specific major within that college. Two hundred forty-eight participants (43.2%) reported that at least one of their majors was in the college that includes the psychology department. A variety of colleges (and therefore majors) were represented because many students who are not psychology majors choose to take psychology courses for general education requirements and/or electives. Participants received extra credit in psychology courses as compensation for their participation.

### Measures

#### Social Anxiety

Two companion measures, the Social Interaction Anxiety Scale (SIAS) and Social Phobia Scale (SPS) [20], were used to assess participants' level of social anxiety. The SIAS assesses social fears related to interacting with others, while the SPS assesses anxiety that is experienced when being observed [20]. Each scale has 20 items rated on a 0 (*not at all characteristic of me*) to 4 (*extremely characteristic of me*) Likert scale, resulting in total scores of 0 to 80 on each measure. Higher scores indicate greater social anxiety. Scores on both scales have demonstrated excellent internal consistency (α = 0.90 to 0.91 in an undergraduate sample) [21] and excellent test-retest reliability (correlations for up to a 12-week period exceeding 0.90) [20]. Internal consistency in this sample was 0.93 for the SIAS and 0.92 for the SPS. On the SIAS, undergraduate students have a mean score of 19.00 (*SD* = 10.10), and individuals with a diagnosis of social anxiety disorder have mean scores between 34.60 (*SD* = 16.40) and 49.99 (*SD* = 15.60) [20], [22]. For the SPS, mean scores are 14.10 (*SD* = 10.20) for undergraduate students and between 32.80 (*SD* = 14.80) and 40.00 (*SD* = 16.00) for clinical samples [20], [22]. When using a cutoff of one standard deviation above the mean from a community sample (i.e., SIAS ≥34 and SPS ≥24), 73% to 82% of individuals with social anxiety disorder were correctly classified [22]. As such, these cut-offs have been used repeatedly to differentiate persons high and low in social anxiety [1], [7], [23].

#### Alcohol Use and ARCs

The mTLFB [1] was used to assess participants' alcohol use and ARCs over the past 30 days. For each drinking occasion, participants reported how many standard drinks they consumed and which aversive consequences they experienced by answering yes or no to a list of 26 ARCs [1]. While Norberg and colleagues [1] listed 24 consequences, two of those items assessed two separate consequences in a single item, and these items were split into separate items as recommended by Schry and Norberg [18]. The original 24 items were shown to reflect four different types of ARCs (i.e., social, physical, personal, and role) in a sample of Australian students [12]. Schry and Norberg [18] examined the factor structure in a sample of college students in the United States; in this study, 22 of the 26 items were found to fit a four-factor structure (i.e., social, physical, personal, and role consequences). Three of the four items not included in the factor structure were not endorsed frequently enough in that sample to be included in the analyses, and one item (“drove a car under the influence”) did not load well on the proposed social factor. Other changes to the factor structure are described in the Statistical Analyses section. The internal consistency of the categories is good, and convergent and concurrent validity have been demonstrated [12], [18].

### Ethics Statements and Study Procedures

All study procedures were approved by the Virginia Tech Institutional Review Board. After participants signed up for the study on the study management website, they were provided with a secure web link where they could read the consent document for this study. All participants implied consent prior to beginning the survey by typing their name and email address at the bottom of a secure website that provided participants with information about the study, including risks and benefits. After implying their consent, participants were directed to a separate, secure website where they completed all study measures. Upon completion of the study measures, participants were provided with a list of local and national health service resources (e.g., counseling centers). To protect participants' privacy, participant codes were used in the dataset rather than identifying information; furthermore, after the conclusion of the study, the key linking participant codes to identifying information was destroyed such that even the authors cannot identify specific participants' data at this time. Therefore, the data file provided (S1 File) contains only deidentified data.

### Statistical Analyses

Following Norberg and colleagues' methodology [1], we conceptualized social anxiety broadly as the presence of either interaction or observational fears. Accordingly, participants were categorized as high in social anxiety if they scored above Heimberg et al.'s cutoffs on either the SIAS (≧34) or SPS (≧24) [22].

Total quantity of alcohol consumed was computed by summing the total number of standard drinks consumed each day for the 30-day assessment period. Additionally, the total number of ARCs experienced in each of the four categories was computed by totaling the number of times each item in that category was endorsed during the 30-day period. Since the number of consequences experienced in a 30-day period is a count variable and therefore positively skewed, Poisson regression analyses were used to examine the relationship between consequences experienced (outcome) and gender and social anxiety (predictors), controlling for total quantity of alcohol consumed. Pairwise comparisons of the marginal means (i.e., the means for each group based on gender and social anxiety level after controlling for total quantity of alcohol consumed) were also conducted. Since Norberg and colleagues' original study [12] was published, three of the four alcohol-related subtypes of the mTLFB have been refined based on findings from factor analysis [18]. The social consequences factor now contains only five of the original eight items and the physical consequences factor contains six of the original seven items, while the personal consequences factor now contains six items (i.e., the original four items plus two additional items that were initially included in the social consequences factor). Thus, all Poisson regression analyses were repeated using the original structure to ensure that any differences obtained with the current sample were due to cultural differences rather than structural differences. All statistical analyses were conducted in PASW 18.0.

## Results

**Figure 1:**
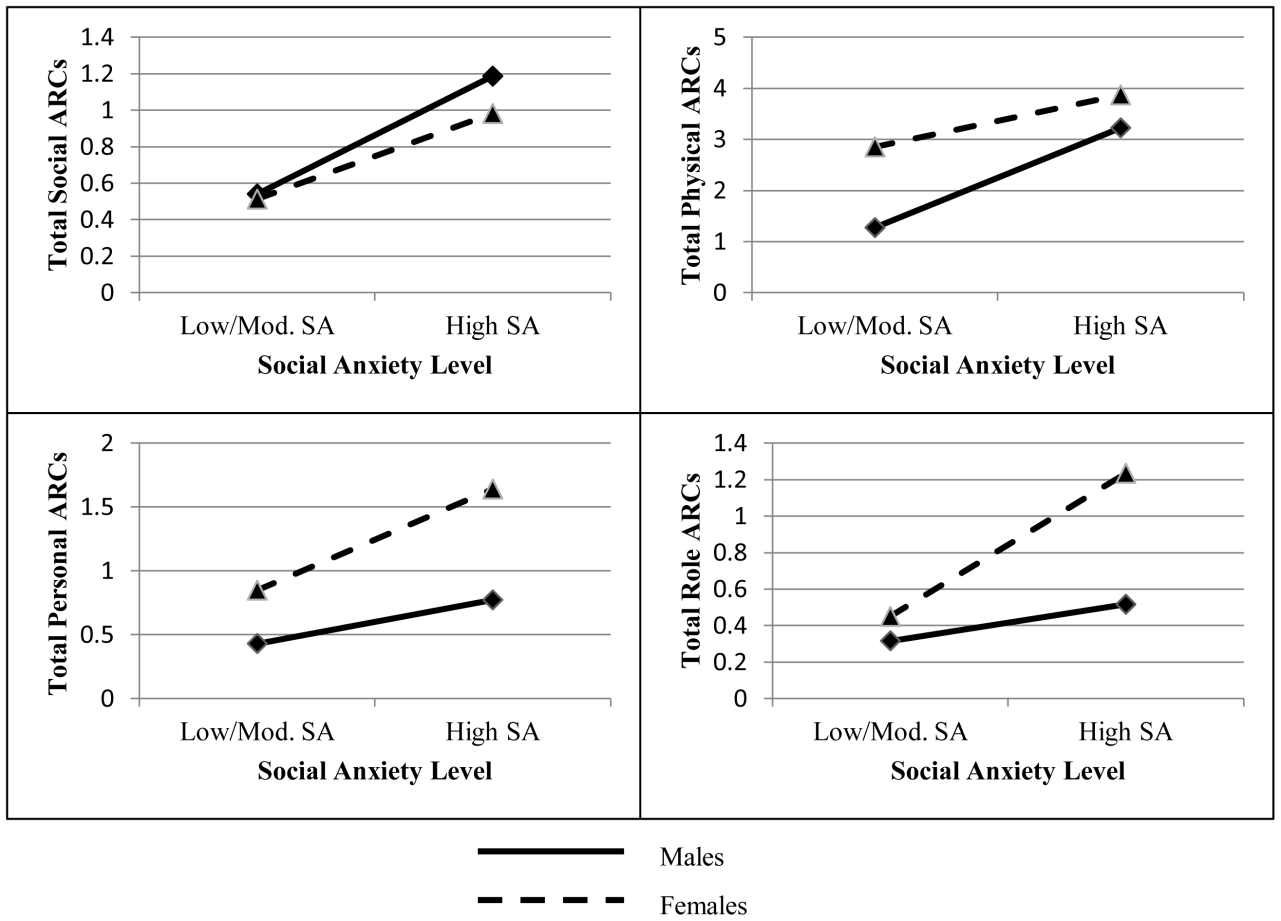
Predicted Values for Each Type of Alcohol-related Consequences. Note: For estimation, quantity was held constant at the sample mean (*M* = 22.65).

One hundred fifty-three participants (26.7% of total sample) were categorized as high in social anxiety. Descriptive statistics are presented in Table 1. Results of the Poisson regression analyses are presented in Table 2. Quantity of alcohol use was a significant, positive predictor of all four categories of ARCs (i.e., social, physical, personal, and role consequences), indicating increased quantity of alcohol use during the 30-day timeframe assessed was associated with increased consequences during that timeframe. The main effect of social anxiety was a significant, positive predictor of all four categories of ARCs. The main effect of gender was significant for three categories of ARCs, such that females were more likely to report physical, personal, and role consequences. The interaction between gender and social anxiety was statistically significant only in the model for physical ARCs. The relationship between social anxiety and physical consequences was stronger for males than females; males with low to moderate social anxiety reported substantially fewer physical ARCs (approximately 1.27) than males high in social anxiety (approximately 3.21). The interaction between gender and social anxiety approached significance in the model for role consequences; this interaction indicated that the relationship between social anxiety and role consequences was stronger for females than males. The predicted number of ARCs for each category is shown in Fig. 1.

**Table 1:** Descriptive statistics.

			Males				Females			
	Overall Sample		High Social Anxiety		Low Social Anxiety		High Social Anxiety		Low Social Anxiety	
	(*n* = 573)		(*n* = 40)		(*n* = 138)		(*n* = 113)		(*n* = 282)	
Variable	*M*	Range	*M*	Range	*M*	Range	*M*	Range	*M*	Range
	*(SD)*		*(SD)*		*(SD)*		*(SD)*		*(SD)*	
SIAS	20.286	0 to 68	35.125	6 to 60	15.710	0 to 33	34.372	11 to 68	14.777	0 to 33
	(12.726)		(11.605)		(8.291)		(11.989)		(8.264)	
										
SPS	14.712	0 to 60	26.875	0 to 56	8.188	0 to 23	30.027	7 to 60	10.043	0 to 23
	(11.629)		(11.590)		(6.147)		(10.201)		(6.212)	
										
Alcohol Use Freq.	3.436	1 to 14	3.125	1 to 7	3.623	1 to 14	2.894	1 to 10	3.606	1 to 13
	(2.214)		(1.897)		(2.500)		(1.877)		(2.201)	
										
Episode Quantity	5.861	1 to 25	7.444	1 to 15	8.012	1 to 24	5.094	1 to 24	4.891	1 to 25
	(3.741)		(3.694)		(4.566)		(3.147)		(2.944)	
										
Quantity	22.646	1 to 207	25.050	1 to 83	33.058	1 to 207	15.620	1 to 89	20.025	1 to 142
	(25.030)		(21.842)		(35.160)		(14.752)		(20.948)	
										
Total Social ARCs	0.759	0 to 12	1.350	0 to 8	0.913	0 to 9	0.903	0 to 11	0.543	0 to 12
	(1.544)		(1.981)		(1.676)		(1.788)		(1.243)	
										
Total Physical ARCs	3.082	0 to 36	3.775	0 to 17	2.457	0 to 25	3.549	0 to 23	3.103	0 to 36
	(4.409)		(4.693)		(3.502)		(4.772)		(4.595)	
										
Total Personal ARCs	1.007	0 to 14	0.900	0 to 5	0.812	0 to 10	1.504	0 to 11	0.918	0 to 14
	(1.859)		(1.392)		(1.601)		(2.391)		(1.763)	
										
Total Role ARCs	0.626	0 to 12	0.600	0 to 4	0.587	0 to 8	1.133	0 to 12	0.486	0 to 9
	(1.454)		(1.057)		(1.413)		(2.098)		(1.139)	
										

Note: SIAS  =  Social Interaction Anxiety Scale; SPS  =  Social Phobia Scale; Alcohol Use Freq.  =  number of days alcohol was consumed in the past 30 days; Episode Quantity  =  average number of standard drinks per drinking episode; Quantity  =  total number of standard drinks during the 30-day period; ARCs  =  alcohol-related consequences.

**Table 2:** Results of poisson regressions.

Dependent Variable	Model *χ^2^(df)*	Predictor	*b*	*SE b*	*χ^2^(1)*	*p*
Social ARCs	236.888*** (4)	Quantity	0.018	0.001	268.150	<0.001
		Gender	−0.054	0.131	0.170	0.680
		Social anxiety	0.785	0.170	21.316	<0.001
		Gender X Social anxiety	−0.136	0.210	0.418	0.518
						
Physical ARCs	955.767*** (4)	Quantity	0.020	0.001	1279.447	<0.001
		Gender	0.806	0.071	130.206	<0.001
		Social anxiety	0.929	0.102	82.277	<0.001
		Gender X Social anxiety	−0.624	0.117	28.459	<0.001
						
Personal ARCs	312.454*** (4)	Quantity	0.020	0.001	387.293	<0.001
		Gender	0.677	0.125	29.527	<0.001
		Social anxiety	0.583	0.199	8.619	0.003
		Gender X Social anxiety	0.075	0.219	0.119	0.730
						
Role ARCs	224.293*** (4)	Quantity	0.020	0.001	244.381	<0.001
		Gender	0.352	0.154	5.218	0.022
		Social anxiety	0.490	0.241	4.126	0.042
		Gender X Social anxiety	0.519	0.267	3.777	0.052
						

Note: ARCs  =  alcohol-related consequences; Quantity  =  total number of standard drinks during the 30-day period; for Gender, male was coded 0 and female was coded 1; for Social anxiety, low to moderate social anxiety was coded 0 and high social anxiety was coded 1; *** *p*<0.001.

The pairwise comparisons revealed that females high in social anxiety reported more social ARCs, physical ARCs, personal ARCs, and role ARCs than males with low to moderate social anxiety and than females with low to moderate social anxiety as well as more physical ARCs, personal ARCs, and role ARCs than males high in social anxiety (all *p*s <0.05). Additionally, males high in social anxiety reported more social ARCs, physical ARCs, and personal ARCs than males with low to moderate social anxiety and more social ARCs than females with low to moderate social anxiety (all *p*s <0.02). Finally, females with low to moderate social anxiety reported more physical ARCs, personal ARCs, and role ARCs than males with low to moderate social anxiety (all *p*s <0.02). The remaining pairwise comparisons were not statistically significant.

The Poisson regression analyses were rerun using the original factor structure [12] to determine whether the differences in findings were due to changes in the factor structure or cross-cultural differences. These results are presented in Table 3. Overall, the pattern of findings is similar to the results presented in Table 2. The predictors identified as statistically significant were identical in both sets of analyses, and significant predictors were in the same direction and of similar magnitude in both analyses.

**Table 3:** Results of poisson regressions using the original factor structure [12].

Dependent Variable	Model *χ^2^(df)*	Predictor	*b*	*SE b*	*χ^2^(1)*	*p*
Social ARCs	297.621*** (4)	Quantity	0.018	0.001	362.978	<0.001
		Gender	0.130	0.114	1.306	0.253
		Social anxiety	0.730	0.157	21.719	<0.001
		Gender X Social anxiety	−0.109	0.187	0.337	0.562
						
Physical ARCs	956.168*** (4)	Quantity	0.020	0.001	1278.922	<0.001
		Gender	0.807	0.071	130.497	<0.001
		Social anxiety	0.935	0.102	83.729	<0.001
		Gender X Social anxiety	−0.629	0.117	29.020	<0.001
						
Personal ARCs	256.487*** (4)	Quantity	0.020	0.001	319.599	<0.001
		Gender	0.666	0.137	23.665	<0.001
		Social anxiety	0.681	0.211	10.442	0.001
		Gender X Social anxiety	−0.028	0.234	0.014	0.904
						
Role ARCs	224.293*** (4)	Quantity	0.020	0.001	244.381	<0.001
		Gender	0.352	0.154	5.218	0.022
		Social anxiety	0.490	0.241	4.126	0.042
		Gender X Social anxiety	0.519	0.267	3.777	0.052
						

Note: ARCs  =  alcohol-related consequences; Quantity  =  total number of standard drinks during the 30-day period; for Gender, male was coded 0 and female was coded 1; for Social anxiety, low to moderate social anxiety was coded 0 and high social anxiety was coded 1; *** *p*<0.001.

## Discussion

The purpose of the current study was to examine the effects of social anxiety and gender on ARCs in a sample of university students in the United States. The hypothesis that social anxiety would be positively related to personal and role ARCs in females was supported, but the effect was not specific to these categories of ARCs or to females. Our hypothesis that males would report more social ARCs than females was not supported, as gender did not predict social ARCs. Controlling for the total number of alcoholic drinks consumed during the 30-day reporting period, social anxiety significantly and positively predicted all four categories of ARCs, and females reported significantly more ARCs in three of the four categories (i.e., role functioning, physical, and personal). The interaction between social anxiety and gender was only significant for physical ARCs, although it approached significance for role ARCs. The relationship between social anxiety and role ARCs was somewhat stronger for females, while the relationship between social anxiety and physical ARCs was significantly stronger for males.

Thus, the current study only replicates a few findings from Norberg and colleagues' Australian study [12]. In both studies, females with high social anxiety reported more personal and role consequences than did females with low to moderate social anxiety. In addition, both studies found that females reported more physical consequences than did males; however, the current study also found that males high in social anxiety reported substantially more physical consequences (approximately 3.21) than did males with low to moderate social anxiety (approximately 1.27). The physical consequences factor only changed minimally since the prior study [18], and the revised factor structure did not influence this finding. Therefore, the differences in study findings are not due to the changes in the factor structure.

There are numerous possible explanations for the differences between the findings in the two studies. First, it is possible that either differences in participant characteristics or differences in cultural factors between the United States and Australia account for study differences. Despite no difference in chronological age between the studies' samples, over two-thirds of participants in the current sample were younger than the legal drinking age, while only 2 participants (1.7% of the sample) were below the legal drinking age in the Norberg and colleagues study [12]. Underage drinkers in the U.S. may experience different consequences relative to peers who are of legal age in Australia, given the tendency of underage drinkers to drink in places that do not require proof of age [15]. Moreover, males in this sample drank substantially more per episode (*M_males - low SA_*  = 8.01, *M_males - high SA_*  = 7.44) than males in the Norberg and colleagues sample (*M_males - low SA_*  = 6.83, *M_males - high SA_*  = 4.24) [1]. These consumption differences might be due to the race/ethnicity differences between the samples. The current sample was predominately Caucasian, whereas the Norberg and colleagues' sample [12] had approximately equal distributions of Caucasian (39.8%) and Asian (44.1%) participants. Facial flushing following alcohol consumption, which is more common in Asian cultures, has been associated with decreased frequency of heavy alcohol consumption and ARCs [24]. The consumption differences might also be due to injunctive norm differences. Injunctive norms relate to how much individuals think their peers approve of alcohol use. Although injunctive norms have been shown to moderate the relationship between social anxiety ARCs among college students [23]. they were not examined in either study.

Finally, differences in the sample sizes between the two studies may also be important to consider. Post-hoc power analyses using the sample size and distribution of social anxiety from the Norberg and colleagues [12] study and the base rates and effect sizes of social anxiety found in this study revealed that Norberg and colleagues' study was adequately powered (i.e., power ≧0.84) to detect the effect of social anxiety on social and physical ARCs, but it was underpowered (i.e., power ≤0.50) to detect the effect of social anxiety on personal and role ARCs found in the present study. The betas for the effect of social anxiety on social and physical ARCs were much smaller in Norberg and colleagues' study than in the present study [12]. Since smaller studies may be subject to sampling biases more so than larger studies, it is possible that Norberg and colleagues' [12] findings are not reliable. In order to detect the smallest effect of social anxiety in this study (i.e., the effect of social anxiety on role ARCs), a sample of 550 participants would be needed to have power of 0.80 based on the characteristics of the sample in this study. Therefore, large samples are suggested for future studies in this area.

This study also examined the effects of gender on types of ARCs. Females endorsed more physical, personal, and role ARCs than males. This finding was surprising, as undergraduate males have been found to engage in risky alcohol use behaviors (including binge drinking), meet diagnostic criteria for an alcohol use disorder, and experience ARCs more commonly than undergraduate females [25]. As can be seen in Table 1, male college students consumed substantially more alcohol than did female college students, yet women experienced more personal, physical, and role consequences, even prior to controlling for alcohol consumption differences. Therefore, these gender differences may reflect the fact that females have a higher vulnerability to the effects of alcohol due to biological reasons [26]. Further research is needed to replicate this effect of gender and, assuming replication, determine why it exists in college students in the United States, as the extant research has yielded inconsistent findings with respect to gender differences in ARCs [8], [10], [11].

Our finding of a high rate of physical consequences among both males and females in this sample is consistent with prior research indicating that short-term physical consequences are common among college students [11], and underscores the importance of assessing physical consequences when examining ARCs in university students. Failure to assess this type of consequence may result in underestimating the problematic effects of alcohol.

### Limitations

The findings presented here should be considered within the context of the limitations. First, all measures were self-report. Self-presentation concerns may affect responses on self-report measures. Additionally, because individuals high in social anxiety are concerned about others negatively evaluating them [27], their endorsement of some ARCs may be affected by a tendency to negatively evaluate their own past behavior or desire to present themselves positively. Furthermore, this study was cross-sectional in design, limiting ability to make inferences about directionality. Also, the sample was drawn from students enrolled in psychology courses during a single semester. Although students with a wide variety of majors take psychology courses for various reasons, the sample may not be representative of all college students. Finally, although the sample was relatively representative of the university population from which it was drawn, most participants were white. Therefore, further research is needed to determine whether these findings generalize to other racial and ethnic backgrounds and to non-college attending individuals.

## Conclusions

Results of the present study are consistent with a growing body of literature suggesting social anxiety is predictive of experiencing ARCs [12], [18]. Females appear to be at increased risk of experiencing physical, personal, and role consequences among college students. Social anxiety is a stronger predictor of physical consequences for males than it is for females. Further research is needed to understand how and why social anxiety influences risk of experiencing ARCs and the potential moderating role of gender. In addition, caution should be taken in extrapolating findings about the relationship between social anxiety, alcohol use, and experience of adverse consequences to other countries and cultures, as factors such as injunctive norms about drinking may play a role.

## Supporting Information

S1 File **Data file for analyses presented in this paper.**
(XLSX)

S2 File **Variable names and coding information for data file.**
(DOCX)
