# Supplementary material for: Gender Matters: The Relationship between Social Anxiety and Alcohol-Related Consequences
Source: PLoS One. 2014 Dec 26;9(12):e115361. doi: 10.1371/journal.pone.0115361 (PMC4277359; doi:10.1371/journal.pone.0115361)
Supplement: S2 File — Variable names and coding information for data file. (DOCX) [file pone.0115361.s002.docx]

**Variable Information**

- Sex
  - 1 = Female
  - 0 = Male
- Age = age in years
- CollYear = Year in College
  - 1 = 1^st^ year
  - 2 = 2^nd^ year
  - 3 = 3^rd^ year
  - 4 = 4^th^ year
  - 5 = 5^th^ year or beyond
- Race/Ethnicity Variables
  - 1 = participant selected that race/ethnicity
  - White = White/Caucasian, non-Hispanic, non-Arab
  - Black = Black/African American, non-Hispanic
  - Hispanic = Hispanic/Latino/a
  - AmerIndian = American Indian/Alaskan Native
  - Arab = Arab/Middle Eastern or Arab American
  - Asian = Asian/Asian-American
  - PacIsl = Pacific Islander
  - NotProv = I do not wish to provide this information
- D1to30DaysDrank = # of days participant drank in past 30 days
- D1to30AveEpQuant = Average # of standard drinks consumed per day in the past 30 days
- D1to30Quant = Total # of standard drinks consumed in the past 30 days
- SIASTotal = Total score on Social Interaction Anxiety Scale
- SPSTotal = Total score on Social Phobia Scale
- SIASCutoff
  - 1 = above clinical cutoff on SIAS
  - 0 = below clinical cutoff on SIAS
- SPS Cutoff
  - 1 = above clinical cutoff on SPS
  - 0 = below clinical cutoff on SPS
- SIASSPSCutoff
  - 1 = above clinical cutoff on either SIAS or SPS
  - 0 = below clinical cutoff on both SIAS and SPS
- SexXSADichInt = Interaction between sex and SIASSPSCutoff
- D1to30TotalARCs = Total # of ARCs in past 30 days
- SocialARCs = Total # of social ARCs in past 30 days
- PhysicalARCs = Total # of physical ARCs in past 30 days
- PersonalARCs = Total # of personal ARCs in past 30 days
- RoleARCs = Total # of role ARCs in past 30 days
- NorSocARCs = Total # of social ARCs (using Norberg et al. [2011] categories) in past 30 days
- NorPhyARCs = Total # of physical ARCs (using Norberg et al. [2011] categories) in past 30 days
- NorPersARCs = Total # of personal ARCs (using Norberg et al. [2011] categories) in past 30 days
- NorRoleARCs = Total # of role ARCs (using Norberg et al. [2011] categories) in past 30 days
